# β-Arrestin-2 attenuates hepatic ischemia-reperfusion injury by activating PI3K/Akt signaling

**DOI:** 10.18632/aging.202246

**Published:** 2020-12-11

**Authors:** Xiaolong Chen, Junbin Zhang, Long Xia, Li Wang, Hui Li, Huilin Liu, Jing Zhou, Zhiying Feng, Hai Jin, JianXu Yang, Yang Yang, Bin Wu, Lei Zhang, Guihua Chen, Genshu Wang

**Affiliations:** 1 Department of Hepatic Surgery, The Third Affiliated Hospital of Sun Yat-Sen University, Guangzhou 510630, Guangdong Province, P. R. China; 2 Guangdong Provincial Key Laboratory of Liver Disease Research, Guangzhou 510630, Guangdong Province, P. R. China; 3 Department of Gastroenterology, The Third Affiliated Hospital of Sun Yat-Sen University, Guangzhou 510630, Guangdong Province, P. R. China; 4 Department of Pathology, The Third Affiliated Hospital of Sun Yat-Sen University, Guangzhou 510630, Guangdong Province, P. R. China; 5 Department of Medical Ultrasonics, Guangzhou First People’s Hospital, The Second Affiliated Hospital of South China University of Technology, Guangzhou 510630, Guangdong Province, P. R. China; 6 Department of Intensive Care Unit, Henan Provincial People's Hospital, Zhengzhou 450003, Henan Province, P. R. China; 7 Department of Biliary-Pancreatic Surgery, The Third Affiliated Hospital, Sun Yat-Sen University, Guangzhou 510630, Guangdong Province, P. R. China

**Keywords:** hepatic ischemia-reperfusion injury, β-Arrestin-2, PI3K/Akt signaling pathway, liver transplantation, hepatic surgery

## Abstract

Hepatic ischemia-reperfusion injury (IRI) remains a common complication during liver transplantation (LT), partial hepatectomy and hemorrhagic shock in patients. As a member of the G protein-coupled receptors adaptors, ARRB2 has been reported to be involved in a variety of physiological and pathological processes. However, whether β-arrestin-2 affects the pathogenesis of hepatic IRI remains unknown. The goal of the present study was to determine whether ARRB2 protects against hepatic IR injury and elucidate the underlying mechanisms. To this end, 70% hepatic IR models were established in *ARRB2* knockdown mice and wild-type littermates, with blood and liver samples collected at 1, 6 and 12 h after reperfusion to evaluate liver injury. The effect of ARBB2 on PI3K/Akt signaling during IR injury was evaluated in vivo, and PI3K/Akt pathway regulation by ARRB2 was further assessed in vitro. Our results showed that ARRB2 knockdown aggravates hepatic IR injury by promoting the apoptosis of hepatocytes and inhibiting their proliferation. In addition, ARRB2 deficiency inhibited PI3K/Akt pathway activation, while the administration of the PI3K/Akt inhibitor PX866 resulted in severe IR injury in mice. Furthermore, the liver-protecting effect of ARRB2 was shown to depend on PI3K/Akt pathway activation. In summary, our results suggest that β-Arrestin-2 protects against hepatic IRI by activating PI3K/Akt signaling, which may provide a novel therapeutic strategy for treating liver ischemia-reperfusion injury.

## INTRODUCTION

Hepatic ischemia-reperfusion injury (IRI) is a common pathophysiological process caused by liver transplantation, partial hepatectomy, hemorrhagic shock, and severe infection. The primary forms of IRI are necrosis and apoptosis [1], and although it is mediated by several factors, the exact disease mechanism remains unclear. Previous studies have focused on the generation and destruction of oxygen free radicals, intracellular calcium overload, neutrophil infiltration, and the release of inflammatory cytokines in IRI [2–5]. In recent years, intracellular and extracellular signal transduction in IRI has become a research hotspot, including phosphoinositide 3-kinase (PI3K)/Akt signaling [6, 7].

PI3K is a member of the phospholipase kinase family, members of which have lipase and protein kinase activities. PI3K/Akt signaling enables cells to respond to various stresses, especially anti-apoptosis and pro-survival responses. PI3K activates a series of downstream protein kinases such as Akt through a second messenger, with Akt phosphorylation being key to activation of the PI3K/Akt signaling pathway. The anti-apoptotic role of phosphorylated Akt involves inhibiting the phosphorylation of downstream pro-apoptotic proteins such as Bad and glycogen synthase kinase 3β (GSK3β), members of the pro-apoptotic factor Bcl family, leading to regulation of cell proliferation, apoptosis, and migration [8–10]. Studies have shown that PI3K/Akt is an important self-protective signaling pathway in IRI [11, 12].

β-Arrestins are regulatory proteins in the G protein-coupled receptors (GPCRs) signaling pathway, family members of which include *β-arrestin-1* and *β-arrestin-2*, which are widely distributed [13]. β-Arrestins play an important role in cell proliferation, differentiation, and apoptosis by taking part in the PI3K/Akt and other signaling pathways through the recruitment of signal transduction molecules and activation of various signal transduction complexes [14, 15]. β-Arrestins can promote or inhibit PI3K/Akt activation under different conditions [16, 17]. For instance, β-Arrestin-2 (encoded by *ARRB2*) inhibits Bad phosphorylation by upregulating PI3K/Akt signaling, which inhibits apoptosis [18]. However, it remains unclear whether ARRB2 affects IRI pathogenesis by regulating the PI3K/Akt signaling pathway.

In the present study, we investigated the role of ARRB2 in a mouse model of hepatic IRI using *ARRB2* wild-type (WT) and knockout (KO) mice and investigated whether it regulates the PI3K/Akt signaling pathway. Our results showed that *ARRB2* knockdown aggravates hepatic IR injury by promoting the apoptosis of hepatocytes and inhibiting their proliferation. In addition, the liver-protecting effect of ARRB2 was shown to depend on PI3K/Akt pathway activation. Our results suggest that β-Arrestin-2 protects against hepatic IRI by activating the PI3K/Akt signaling pathway, which may provide a novel therapeutic strategy for treating liver ischemia-reperfusion injury.

## RESULTS

### ARRB2 protects against hepatic ischemia-reperfusion injury in mice

**Figure 1:**
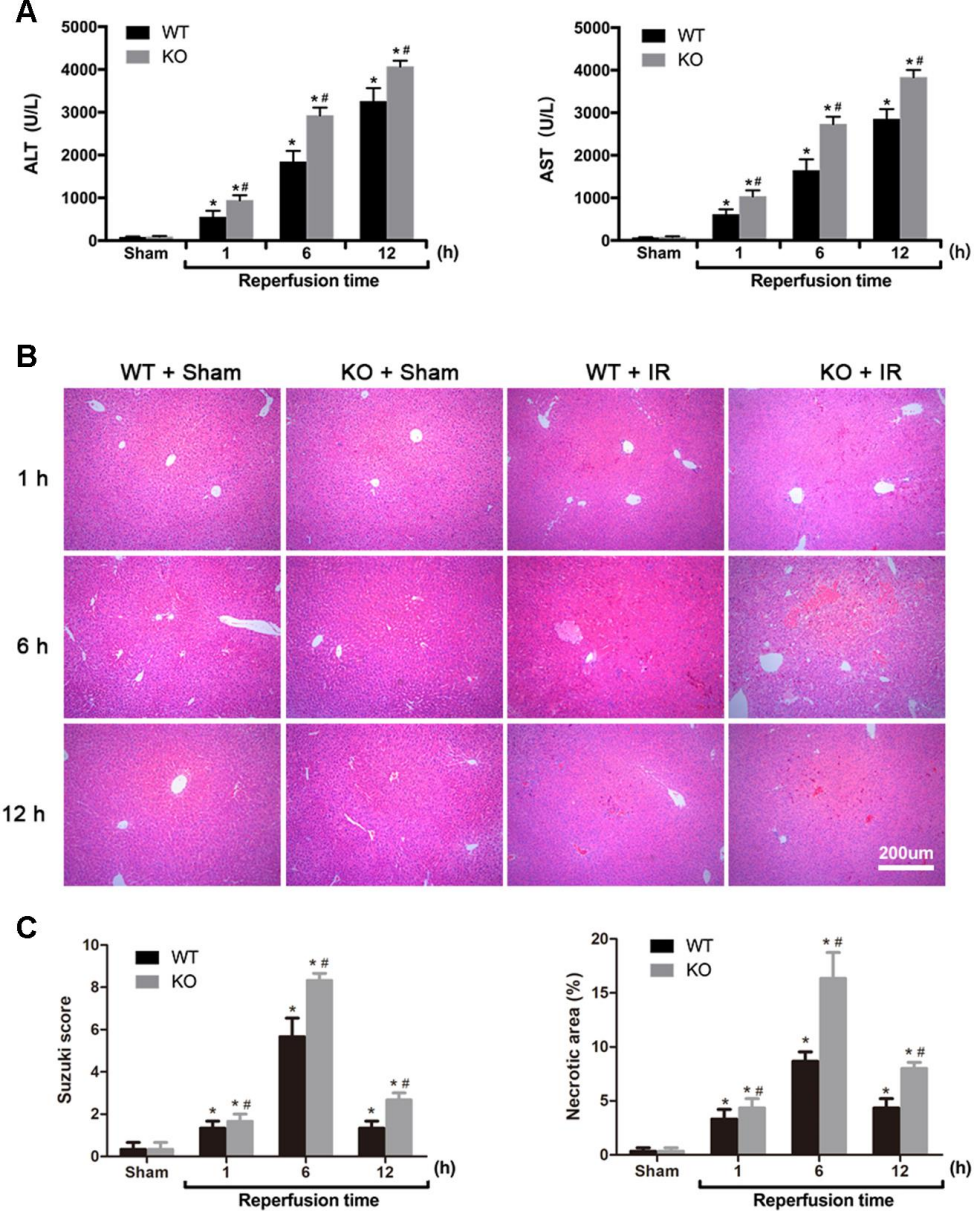
**ARRB2 protects against hepatic ischemia-reperfusion injury in mice.** (**A**) Analysis of serum ALT and AST levels in Sham, WT + IR and KO + IR groups. (**B**) Representative H&E staining of liver tissues (original magnification, 100×). (**C**) Bar graphs show Suzuki score and necrosis area revealed by H&E staining. The data are presented as the Mean ± SD, n = 6. ^*^ *P* < 0.01, compared to Sham group, ^#^ *P*< 0.01, compared to WT + IR group by Student's-t test.

To investigate the role of ARRB2 in hepatic IRI, hepatic histopathologic injuries and serum ALT and AST levels in WT and *ARRB2* KO mice were examined. Compared with those observed in the Sham group, post-IRI ALT and AST levels were significantly increased in the IR group (all *P* < 0.01). ALT and AST levels were significantly higher in the KO + IR group than those observed in the WT + IR group (all *P* < 0.01), whereas no significant difference was observed between the Sham groups (all *P* > 0.05; Figure 1A). H&E staining results revealed normal hepatic cell morphology and hepatic lobular structures at all time points in the Sham groups. In contrast, light to moderate hepatocyte swelling and slightly ectatic liver sinuses were observed in the liver 1 h after reperfusion in the WT + IR and KO + IR groups. Severe hepatocyte swelling, inflammatory cell infiltration, and flaky necrosis were observed 6 h after reperfusion in both groups. Liver injury was more severe and widespread at all time points after reperfusion in the KO + IR group compared to that observed in the WT + IR group (Figure 1B). Consistent with liver injury indicated by histological assessments, the necrotic areas and Suzuki scores were significantly increased in KO + IR groups compared to those observed in the WT + IR groups (Figure 1C). Thus, the above findings suggested that ARRB2 attenuates hepatic IRI in mice.

### ARRB2 inhibits hepatocyte apoptosis in hepatic ischemia-reperfusion injury

**Figure 2:**
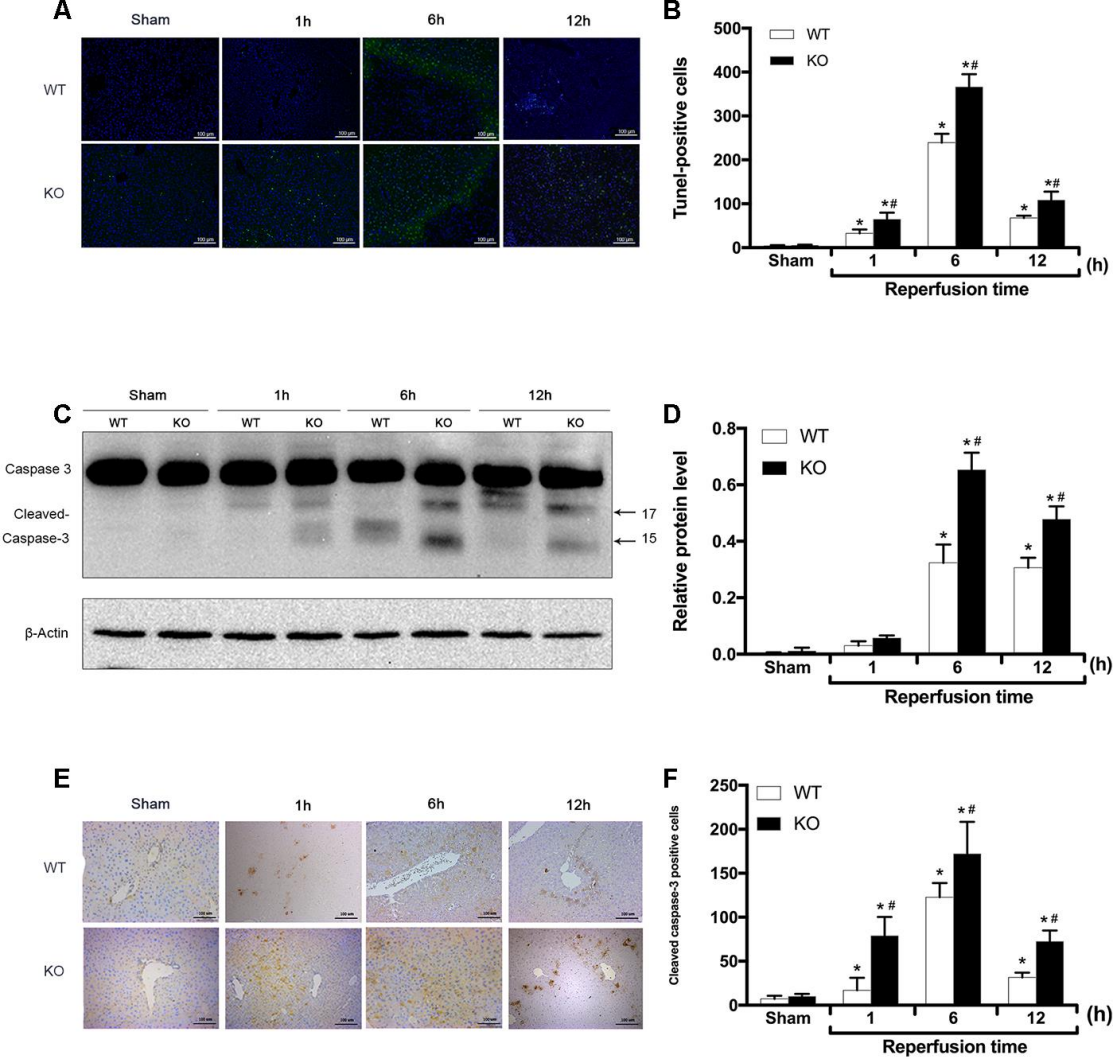
**ARRB2 inhibits hepatocyte apoptosis in hepatic ischemia-reperfusion injury.** (**A**) and (**B**), TUNEL staining of the liver tissue and quantitative analysis of TUNEL positive cells (green) in Sham, WT + IR and KO + IR groups. (**C**) and (**D**), Cleaved Caspase-3 protein level in liver tissue detected by Western Blotting. β-actin was used as an internal control. (**E**) and (**F**), The Immunohistochemical staining of Cleaved Caspase-3 in liver tissues. The data are presented as the Mean ± *SD*, *n* = 6. ^*^P< 0.01, compared to Sham group, ^#^ *P* < 0.01, compared to WT + IR group by Student's-t test.

As apoptosis is the primary manifestation of hepatic IRI, the apoptotic rate in liver tissues was determined via TUNEL staining and the detection of cleaved caspase-3 expression. As shown in Figure 2A, 2B, the number of TUNEL-positive cells did not significantly differ between the Sham groups (*P* > 0.05). However, compared with that observed in the Sham groups, the number of TUNEL-positive cells in the WT + IR and KO + IR groups was significantly increased 1 h after reperfusion, peaked at 6 h, then decreased slightly by 12 h (*P* < 0.01 for each time point; Figure 2A, 2B). Overall, at each time point after reperfusion, the number of TUNEL-positive cells in the KO + IR group was significantly higher than that observed in the WT + IR group (*P* < 0.05; Figure 2A, 2B). Consistent with the TUNEL assay results, the immunoblotting analysis showed that IR injury induced higher cleaved-caspase-3 expression in the KO + IR groups than that observed in the WT + IR groups (*P* < 0.05; Figure 2C, 2D). Furthermore, the IHC staining results also revealed significantly more cleaved caspase 3-positive cells in the KO + IR group than that was observed in the WT + IR group (*P* < 0.05; Figure 2E, 2F). These results showed that postreperfusion hepatocyte apoptosis was more severe in the liver tissues of *ARRB2* KO mice than in WT mice, suggesting that ARRB2 inhibits hepatocyte apoptosis resulting from hepatic ischemia-reperfusion.

### ARRB2 promotes liver proliferation during hepatic ischemia-reperfusion

**Figure 3:**
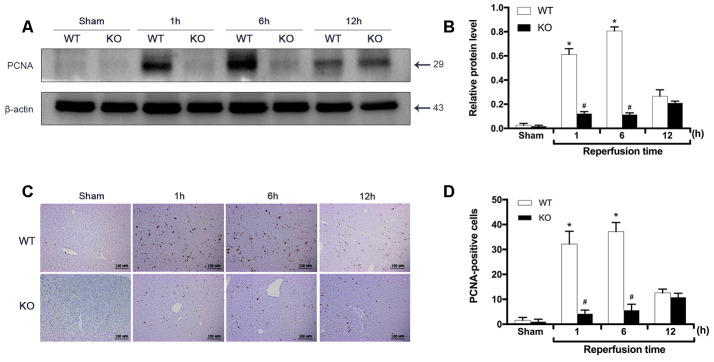
**ARRB2 promotes liver proliferation during hepatic ischemia-reperfusion.** (**A**) and (**B**), PCNA protein level in liver tissue detected by Western Blotting in Sham, WT + IR and KO + IR groups. (**C**) and (**D**), Immunohistochemical staining of PCNA in liver tissues (×200). The data are presented as the Mean ± *SD*, *n* = 6. ^*^ *P*< 0.01, compared to Sham group, ^#^ *P* < 0.01, compared to WT + IR group by Student’s-t test.

To investigate the effect of ARRB2 on liver proliferation during IRI, the expression of PCNA between groups was measured by Western blot and IHC staining analyses. The Western blot results showed no significant difference in PCNA expression between the Sham groups (*P* > 0.05). In contrast, PCNA expression was dramatically increased in the WT+IR group compared to that observed in the KO+IR group 1 and 6 h after reperfusion. Interestingly, PCNA levels significantly increased at the 12 h time point in the KO+IR group, with no difference observed between the KO and WT mice subjected to IR injury (*P* < 0.05; Figure 3A, 3B). Consistent with these immunoblotting results, the PCNA levels detected by IHC staining showed similar changing trends (*P* < 0.05; Figure 3C, 3D). These findings indicated that ARRB2 promotes the proliferation of hepatocytes in mice suffering from hepatic ischemia-reperfusion.

### ARRB2 deficiency inhibits PI3K/Akt signaling activation

**Figure 4:**
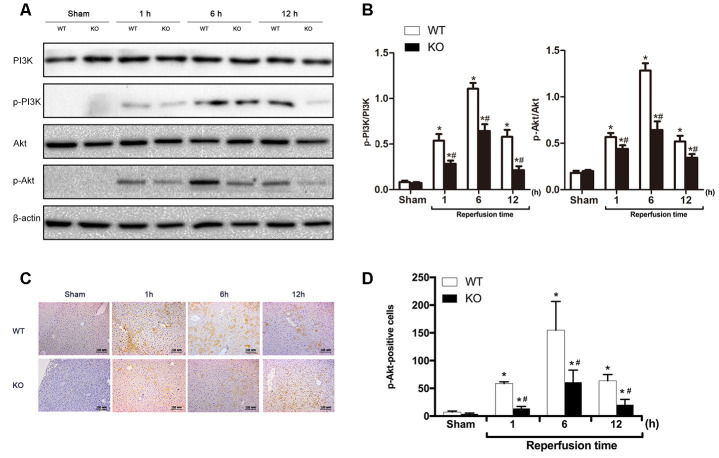
**ARRB2 deficiency inhibits PI3K/Akt signaling activation.** (**A**) PI3K, p-PI3K, AKT, p-AKT protein levels in liver tissue detected by Western Blotting in Sham, WT + IR and KO + IR groups. (**B**) Quantitative analysis of p-PI3K/PI3K levels and p-Akt/Akt levels. (**C**) and (**D**), Immunohistochemical staining of p-Akt in liver tissues (×200). The data are presented as the Mean ± *SD*, *n* = 6. * *P*< 0.01, compared to Sham group, ^#^*P* < 0.01, compared to WT + IR group by Student’s-t test.

Activated PI3K/Akt signaling can promote cell survival and inhibit apoptosis, and ischemic preconditioning has been shown to protect mice against hepatic IRI by activating the PI3K/Akt signaling pathway [19]. Therefore, we subsequently examined whether PI3K/Akt signaling was affected by ARRB2 during IR damage. The western blot results showed that compared with the markedly increased p-PI3K and p-AKT levels detected 6 and 12 h post reperfusion, PI3K/Akt signaling was significantly inhibited in *ARRB2* KO mice during I/R-induced liver injury (*P* > 0.05; Figure 4A, 4B). Then, the level of phosphorylated Akt (p-Akt), the key downstream protein of the activated PI3K/Akt signaling pathway, was further measured by IHC staining. The results revealed that significantly more p-Akt-positive cells were present in the WT + IR group compared with that detected in the KO + IR group at each time point (*P*< 0.05; Figure 4C, 4D). Considering that ARRB2 has been shown to inhibit the apoptosis of hepatocytes and promote their proliferation in mice suffering from hepatic ischemia reperfusion, our results suggested that ARRB2 may protects mice against hepatic IRI by activating PI3K/Akt signaling.

### Inhibition of PI3K/Akt signaling aggravates hepatic ischemia-reperfusion injury in mice

**Figure 5:**
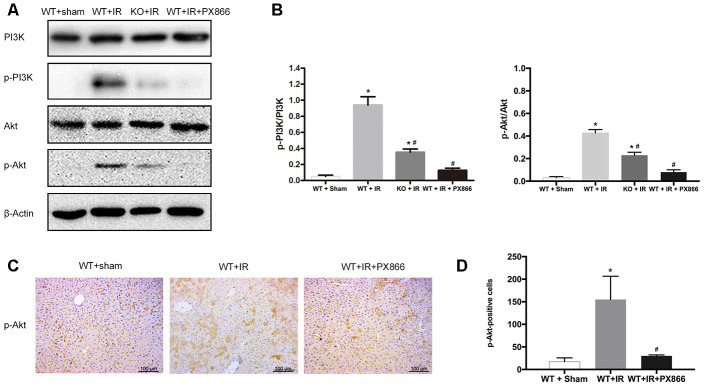
**PX866 inhibits PI3K/Akt signaling activation.** (**A**) PI3K, p-PI3K, AKT, p-AKT protein levels at 6 h after reperfusion detected by Western Blotting in Sham, WT + IR, KO + IR, and WT + IR + PX866 groups. (**B**) Quantitative analysis of p-PI3K/PI3K levels and p-Akt/Akt levels. (**C**) and (**D**), Immunohistochemical staining of p-Akt in liver tissues (×200). The data are presented as the Mean ± *SD*, *n* = 6. ^*^P<0.01, compared to Sham group, ^#^ *P*< 0.01, compared to WT + IR group by Student’s-t test.

**Figure 6:**
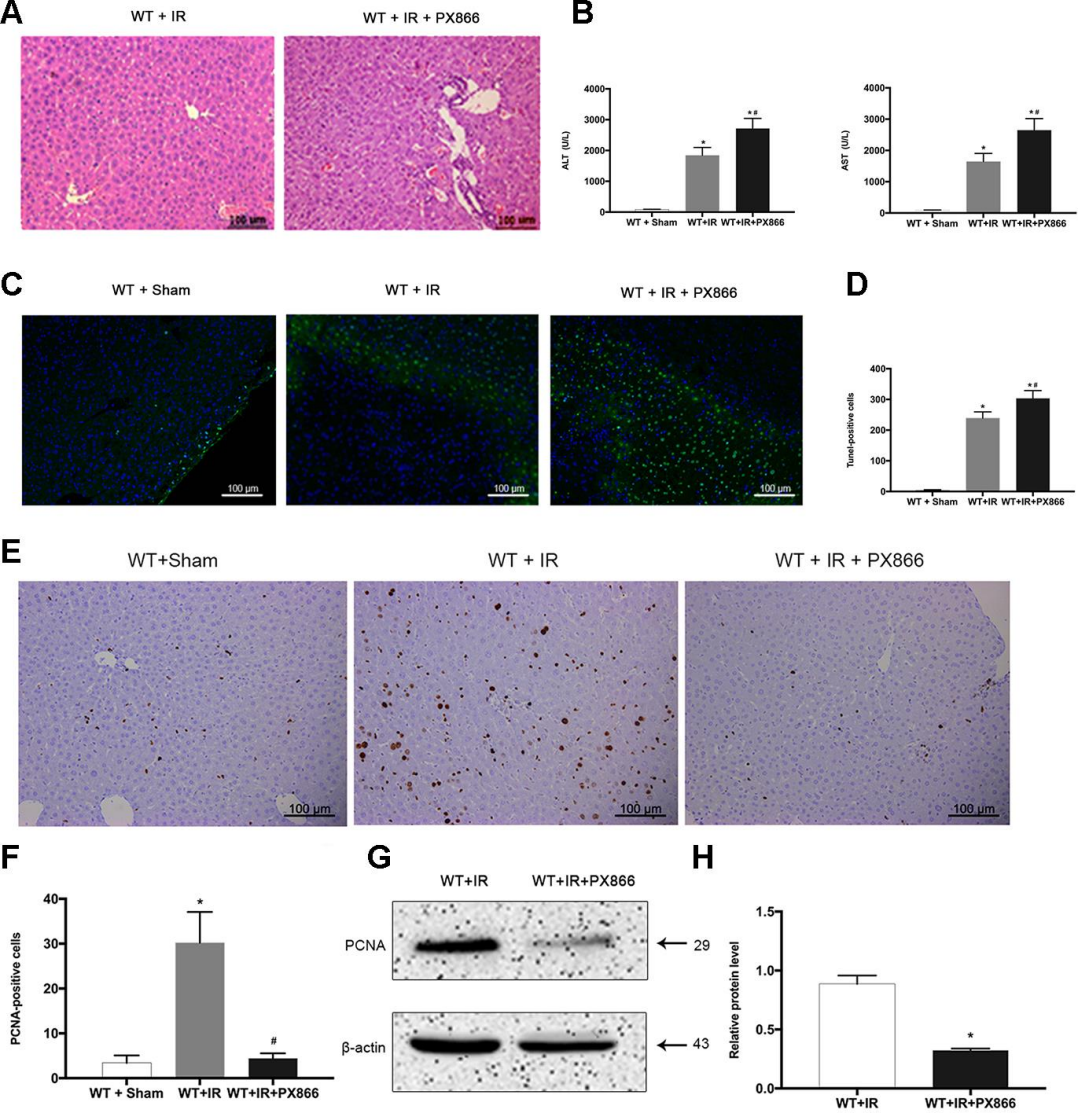
**Inhibition of PI3K/Akt signaling aggravates hepatic ischemia-reperfusion injury in mice.** (**A**) Representative H&E staining of liver tissues at 6 h after reperfusion in WT + IR and WT + IR + PX866 groups (×200). (**B**) Analysis of serum ALT and AST levels (**C**) and (**D**), TUNEL positive cells (green) at 6 h after reperfusion. (**E**) and (**F**), PCNA protein in liver tissues detected by immunohistochemical staining (×200). (**G**) and (**H**), PCNA protein in liver tissue 6 h after reperfusion detected by Western Blotting. The data are presented as the Mean ± *SD*, *n* = 6. ^*^P<0.01, compared to Sham group, ^#^ *P*< 0.01, compared to WT + IR group by Student’s-t test.

To further verify the protective effect of ARRB2-activated PI3K/Akt signaling in mice with hepatic IRI, the PI3K/Akt signaling inhibitor PX866 was intraperitoneally administered to WT mice 1 h before ischemia. The level p-Akt 6 h after reperfusion was detected by western blotting and IHC staining. p-PI3K and p-Akt levels were significantly decreased in the WT + PX866 group compared to that observed in the control group (*P* < 0.01; Figure 5A, 5B), confirming the successful suppression of PI3K/Akt signaling. Furthermore, PX866 administration resulted in severe liver injury in WT mice 6 h after reperfusion (Figure 5C, 5D), as revealed by histological observations and increased serum levels of ALT and AST (*P* < 0.05, Figure 6A, 6B). Moreover, the number of TUNEL-positive cells was also significantly increased (*P* < 0.01, Figure 6C, 6D), and PCNA protein levels were significantly decreased in PX866-pretreated WT mice (*P* < 0.01, Figure 6E–6H). These results indicated that inhibiting PI3K/Akt signaling promotes the apoptosis of hepatocytes and suppresses their proliferation during hepatic ischemia-reperfusion in mice.

### ARRB2 protects hepatocytes against IR injury by activating the PI3K/Akt pathway

**Figure 7:**
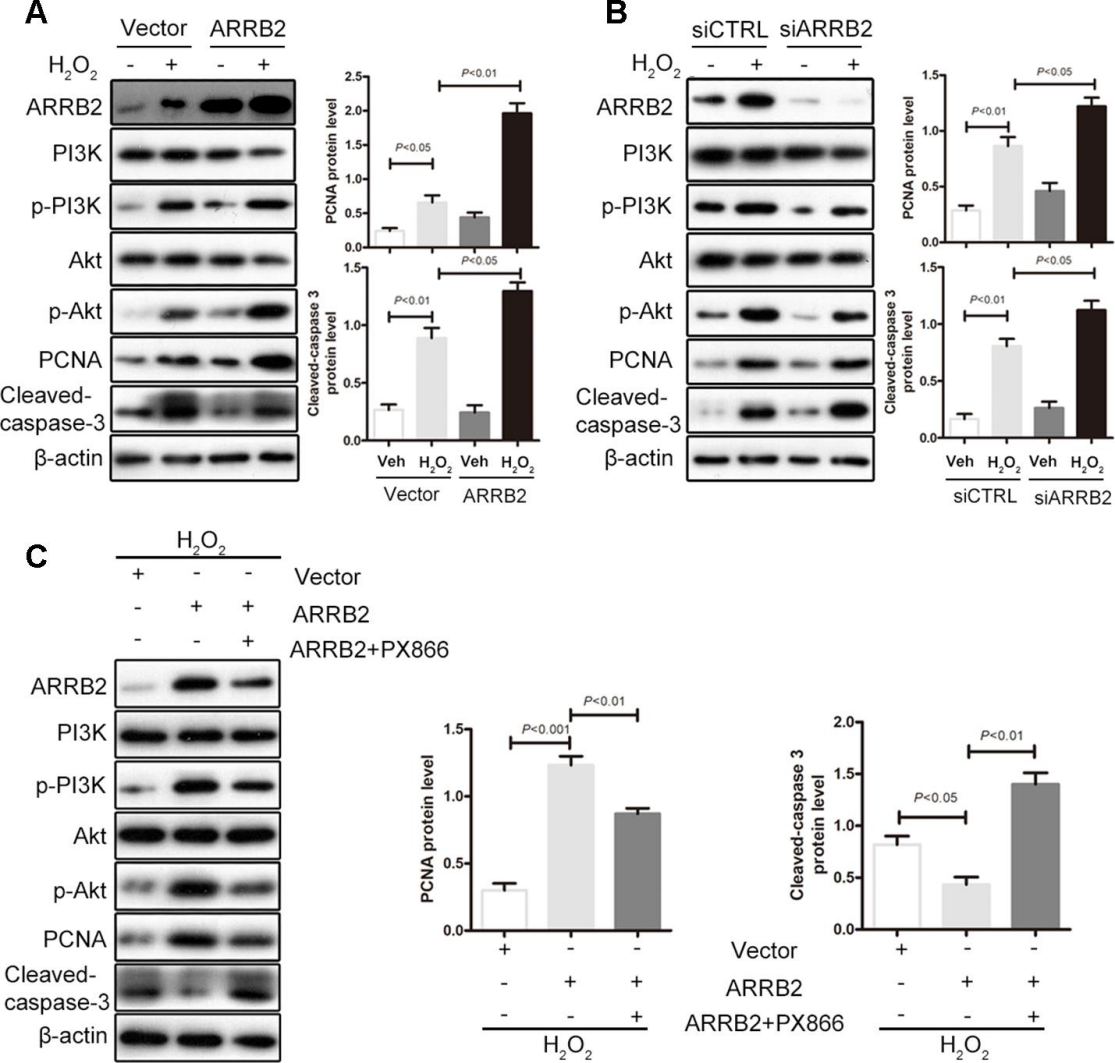
**ARRB2 protects hepatocytes against IR injury by activating the PI3K/Akt pathway.** L02 cells were treated with H_2_O_2_ to mimic oxidative stress during ischemia reperfusion. (**A**) Overexpress of ARRB2 marked increases in PI3K and Akt phosphorylation and PCNA expression as well as notably decreased cleaved caspase-3 levels. (**B**) Knockdown of ARRB2 significantly inhibited PI3K/Akt phosphorylation, inhibited PCNA expression and increased cleaved caspase-3 expression. (**C**) PI3K/Akt inhibitor PX866 significantly inhibited ARRB2 dinduced-PI3K/Akt phosphorylation, downregulated PCNA expression and upregulated cleaved caspase-3 expression. The data are presented as the Mean ± *SD*, *n* = 3. *P*<0.05, *P*< 0.01, *P*< 0.001using Student’s-t test.

Based on the observations described above, we further assessed whether the liver-protecting effect of ARRB2 depends on PI3K/Akt pathway activation. Subsequently, normal hepatocyte L02 cells were treated with H_2_O_2_ to mimic oxidative stress during ischemia reperfusion and then transfected with an ARRB2 expression plasmid. Compared to that observed in the control group, PI3K/Akt signaling was activated during oxidative stress injury. Furthermore, in ARRB2-overexpressing cells, marked increases in PI3K and Akt phosphorylation and PCNA expression as well as notably decreased cleaved caspase-3 levels were observed compared to that detected in control cells (Figure 7A). In contrast, endogenous ARRB2 knockdown using siRNA significantly reduced PI3K/Akt phosphorylation, inhibited PCNA expression and increased cleaved caspase-3 expression (Figure 7B). Furthermore, when LO2 cells were treated with the PI3K/Akt inhibitor PX866 after ARRB2 plasmid transfection and observed that PI3K/Akt phosphorylation was fully restrained, PCNA expression was markedly reduced and cell apoptosis was significantly upregulated (Figure 7C). Taken together, these results suggest that ARRB2 protects hepatocytes against IR injury by activating the PI3K/Akt pathway.

## DISCUSSION

Apoptosis is the primary manifestation of hepatic IRI, but its underlying mechanism remains unclear [20]. Multiple signal transduction pathways including PI3K/Akt, have been demonstrated to play important roles in hepatic IRI [21, 22]. Bcl-2 family proteins promote the mitochondrial release of cytochrome C and apoptosis-related factors such as caspase-3, which induces apoptosis. Bad is one such pro-apoptotic protein of the Bcl-2 family [23]. The PI3K/Akt signaling pathway promotes 14-3-3 protein chelation in the cytoplasm by phosphorylating the Serl36 residue of Bad, thereby terminating the Bad-mediated antagonism of Bcl-2 and Bcl-xL on the mitochondrial membrane and restoring their anti-apoptotic function [9, 10, 24]. ARRB2 prevents Bad phosphorylation by upregulating PI3K/Akt and other signaling pathways, thereby inhibiting apoptosis [18]. Therefore, we speculated that ARRB2 may affect ischemia-reperfusion-mediated hepatocyte apoptosis and subsequent repair by regulating the PI3K/Akt signaling pathway.

Our findings revealed significantly higher serum ALT and AST levels and more severe pathological damage in *ARRB2* KO mice than that observed in WT mice after hepatic ischemia-reperfusion, suggesting that ARRB2 protects mice against hepatic IRI.

IRI is closely associated with apoptosis. For instance, tissue necrosis induced by the initiation of apoptosis and subsequent cascade reactions were shown to be involved in liver injury [25, 26]. Caspase-3 is a caspase family enzyme that plays an important role in cellular apoptosis. Thus, activated cleaved caspase-3 is a marker of apoptosis [27]. In the hepatic ischemia-reperfusion injury mouse models, we observed significantly greater apoptosis in the liver tissues of *ARRB2* KO mice compared with WT mice after hepatic ischemia-reperfusion, as detected by TUNEL staining and increased cleaved caspase-3 expression. Furthermore, PCNA expression was higher in WT mice than in *ARRB2* KO mice. These results suggest that ARRB2 alleviates hepatic IRI by inhibiting hepatocellular apoptosis and promotes repair by enhancing hepatocellular proliferation.

Activated PI3K/Akt signaling inhibits apoptosis and promotes cell survival [28]. Zhang et al. [29] observed that prolactin D1 alleviates hepatic IRI by activating the PI3K/Akt signaling pathway in rats. Similarly, Rao et al. [30] showed that activated transcription factor 3-mediated Nrf2/HO-1 signaling activates PI3K/Akt signaling, thereby inhibiting Toll-like receptor 4/nuclear factor (NF)-kB-regulated inflammatory mediators and alleviating hepatic IRI in mice. ARRB2 has been shown to inhibit phosphorylation of the pro-apoptotic protein Bad by upregulating PI3K/Akt signaling, which inhibits apoptosis [18]. However, whether β-arrestin-2 alleviates hepatic IRI by regulating the PI3K/Akt signaling pathway has remained unclear.

Akt is the direct downstream target protein of PI3K, and phosphorylated Akt activates anti-apoptotic mechanisms, glucose metabolism, and protein synthesis, which promote cell survival and proliferation. We observed increased levels of p-PI3K, p-Akt and the hepatocyte proliferation-related marker PCNA in liver tissues after ischemia-reperfusion in mice, indicating that ischemia-reperfusion may activate the PI3K/Akt signaling pathway and its downstream target proteins of Akt to initiate liver repair following injury. We also observed a higher p-Akt and PCNA levels in the liver tissues of WT mice than in *ARRB2* KO mice. Moreover, inhibition of PI3K/Akt signaling by PX866 increased hepatocyte apoptosis and aggravated liver injury after hepatic ischemia-reperfusion in WT mice. Furthermore, the in vitro results confirmed that the liver-protecting effect of ARRB2 depends on PI3K/Akt signaling activation. Taken together, these results indicate that ARRB2 inhibits hepatocyte apoptosis and enhances hepatocyte proliferation after ischemia-reperfusion in mice by activating the PI3K/Akt signaling pathway.

In summary, the results of our present study indicated that ARRB2 protects mice against hepatic IRI by inhibiting the apoptosis of hepatocytes and promoting their proliferation through PI3K/Akt signaling pathway activation. These results reveal a novel protective role of ARRB2 in hepatic IRI and may provide novel therapeutic strategy for treating liver ischemia-reperfusion injury.

## MATERIALS AND METHODS

### Mice

WT mice and *ARRB2* KO littermates (6-8 weeks old) in a C57BL/6 background were generated from heterozygote intercrosses (kindly provided by Dr. R. J. Lefkowitz, Duke University Medical Centre, Durham, NC, USA). Genotyping was performed by PCR as described in a previous study [31]. The mice were housed at the Institute of Laboratory Animal Science, the Third Affiliated Hospital of Sun Yat-Sen University. All mice were allowed access to water and chow *ad libitum* and maintained with a 12/12-h light/dark cycle. All the experimental procedures were approved by the Institutional Animal Care and Use Committee at The Third Affiliated Hospital of Sun Yat-Sen University.

### Hepatic ischemia-reperfusion injury mouse models

Nonlethal models of segmental (70%) hepatic warm ischemia and reperfusion were established in WT and *ARRB2* KO mice as previously described [32, 33]. Briefly, the portal triad (including portal vein, hepatic artery, and bile duct) to the left and median liver lobes was occluded with a microvascular clamp for 90 min, and reperfusion was then initiated by removal of the clamp. Animals were sacrificed at 1, 6, and 12 h after reperfusion, after which liver and blood samples were collected. Sham-operated controls received the same procedures without vascular occlusion. The PI3K/Akt inhibitor PX866 (3 mg/kg; Cell Signaling Technology, Danvers, MA, USA) was intraperitoneally administered to WT mice 1 h before ischemia.

### Liver function assay and histologic examination

Serum alanine aminotransferase (ALT) and aspartate aminotransferase (AST) levels were measured as indicators of liver injury with an autoanalyzer (ANTECH Diagnostics, Los Angeles, CA, USA). Formalin-fixed liver tissues were embedded in paraffin and cut into 4-μm-thick sections. The sections were then stained with hematoxylin-eosin (H&E), and slides were blindly analyzed for inflammation and tissue injury using modified Suzuki’s criteria [32]. The necrosis area and Suzuki score of all liver sections were independently assessed by two pathologists.

### Immunohistological and TUNEL staining

Immunohistological staining of liver sections was performed according to the manufacturer’s instructions. In brief, after antigen retrieval using an ethylenediaminete traacetic acid antigen retrieval solution (pH 9.0), the slides were incubated with primary antibodies against proliferating cell nuclear antigen (PCNA), cleaved caspase-3, and phospho-Akt (Cell Signaling Technology). Subsequently, the slides were stained with the ABC staining system (Santa Cruz Biotechnology, Santa Cruz, CA, USA) and then counterstained with hematoxylin. The terminal deoxynucleotidyl transferase-mediated dUTP-digoxigenin nick end labeling (TUNEL) assay was performed using an in situ cell death detection kit (Roche, Basel, Switzerland) according to the manufacturer’s instructions. Nuclei with clear green staining were regarded as TUNEL-positive apoptotic cells. The numbers of positive cells per 1000 cells of each sample are reported as the means ± standard deviation (SD), after which labeled cells in 10 fields of view were counted (magnification, 200×).

### Western blot analysis

Western blot analysis of whole cell lysates from liver tissues was performed was performed according to the manufacturer’s instructions. Equal concentrations of protein from liver samples were separated by 12% sodium dodecyl sulfate polyacrylamide gel electrophoresis and ten transferred to nitrocellulose membranes (Bio-Rad, Hercules, CA, USA). Subsequently, the membranes were incubated overnight with antibodies against the following proteins: PCNA, PI3K, p-PI3K, p-Akt, Akt, cleaved caspase-3, caspase-3, and β-actin (Cell Signaling Technology, Danvers, MA, USA ).

### Cell culture and treatment

The normal hepatocyte cell line L02 was purchased from the American Type Culture Collection, and cells were cultured in DMEM supplemented with 10% FBS. The cells were treated with H_2_O_2_ (100 mM) for 1 h to mimic oxidative stress injury during reperfusion. In some experiments, L02 cells were treated with 10 μmol/l of the PI3K inhibitor PX866 for 2 h (CST, USA).

### Short interfering RNA transfection assay

Small interfering RNA (siRNA) transfection was performed according to the instructions provided by Gene Pharma, China. In brief, cells were seeded in six-well plates and transfected with ARRB2 RNA oligos (20 mM) using a kit (Gene Pharma, China). Western blot analysis was used to assess ARRB2 expression after transfection.

### Statistical analysis

Qualitative data, including IHC staining and Western blot results, are representative of at least three independent experiment. Quantitative data are presented as the means ± SD. Statistical differences between multiple groups were compared by one-way analysis of variance. Statistical differences between two groups were determined by two-tailed unpaired Student’s t-tests. *P* < 0.05 was considered statistically significant. All analyses were performed using SPSS 20.0 (Armonk, NY, USA).
